# The impact of comorbid disease history on all-cause and cancer-specific mortality in myeloid leukemia and myeloma – a Swedish population-based study

**DOI:** 10.1186/s12885-015-1857-x

**Published:** 2015-11-05

**Authors:** Mohammad Mohammadi, Yang Cao, Ingrid Glimelius, Matteo Bottai, Sandra Eloranta, Karin E. Smedby

**Affiliations:** 1 Division of Epidemiology, Institute of Environmental Medicine, Karolinska Institutet, Stockholm, Sweden; 2 Institute of Environmental Medicine, Unit of Biostatistics, Division of Epidemiology, Karolinska Institutet, Stockholm, Sweden; 3 Department of Medicine, Clinical Epidemiology Unit, Solna, Karolinska Institutet, Karolinska University Hospital, Stockholm, Sweden; 4 Department of Immunology, Genetics and Pathology, Unit of Oncology, Uppsala University, Uppsala, Sweden; 5 Hematology Center, Karolinska University Hospital, Stockholm, Sweden

## Abstract

**Background:**

Comorbidity increases overall mortality in patients diagnosed with hematological malignancies. The impact of comorbidity on cancer-specific mortality, taking competing risks into account, has not been evaluated.

**Methods:**

Using the Swedish Cancer Register, we identified patients aged >18 years with a first diagnosis of acute myeloid leukemia (AML, *N* = 2,550), chronic myeloid leukemia (CML, *N* = 1,000) or myeloma (*N* = 4,584) 2002–2009. Comorbid disease history was assessed through in- and out-patient care as defined in the Charlson comorbidity index. Mortality rate ratios (MRR) were estimated through 2012 using Poisson regression. Probabilities of cancer-specific death were computed using flexible parametric survival models.

**Results:**

Comorbidity was associated with increased all-cause as well as cancer-specific mortality (cancer-specific MRR: AML = 1.27, 95 % CI: 1.15–1.40; CML = 1.28, 0.96–1.70; myeloma = 1.17, 1.08–1.28) compared with patients without comorbidity. Disorders associated with higher cancer-specific mortality were renal disease (in patients with AML, CML and myeloma), cerebrovascular conditions, dementia, psychiatric disease (AML, myeloma), liver and rheumatic disease (AML), cardiovascular and pulmonary disease (myeloma). The difference in the probability of cancer-specific death, comparing patients with and without comorbidity, was largest among AML patients <70 years, whereas in myeloma the difference did not vary by age among the elderly. The probability of cancer-specific death was generally higher than other-cause death even in older age groups, irrespective of comorbidity.

**Conclusion:**

Comorbidities associated with organ failure or cognitive function are associated with poorer prognosis in several hematological malignancies, likely due to lower treatment tolerability. The results highlight the need for a better balance between treatment toxicity and efficacy in comorbid and elderly AML, CML and myeloma patients.

**Electronic supplementary material:**

The online version of this article (doi:10.1186/s12885-015-1857-x) contains supplementary material, which is available to authorized users.

## Background

Survival from myeloid leukemia and myeloma has improved during recent decades, but still more than 150,000 patients died of these malignancies worldwide in 2012 [1, 2]. The incidence of most hematological malignancies increases with age, as does the prevalence of many non-malignant chronic disorders [3]. Although patients with comorbid disease can be expected to have a lower tolerance to standard chemotherapy-based regimens used to treat hematological malignancies, evidence to guide clinical decision making in these situations is poor. Clinical trials used as a basis for general treatment recommendations provide insufficient guidance for the treatment of patients with severe comorbid disease since these patients are often underrepresented.

Among cancer patients in general, severe comorbid disease increases overall mortality [4], but results for cancer-specific mortality are mixed [5–8]. In hematological malignancies, multiple studies have established comorbid disease overall as an independent predictor of all-cause mortality [9–14], especially among patients eligible for hematopoietic stem cell transplantation [15–17]. However, most of these studies exclude patients over 70 years of age. Low socioeconomic status has been associated with elevated mortality among patients with acute myeloid leukemia (AML) and multiple myeloma [18], potentially mediated through a more advanced disease at the time of diagnosis, and/or through comorbid disease. Another potential mediating factor between low SES and mortality is access to health care services, although the Swedish social security system does offer universal access of care.

For most hematological malignancies, it is not clear to what extent specific comorbid diseases affect overall and cancer-specific survival, and if effects differ by age. Such estimations could guide clinicians in choosing the most optimal treatments for these patients. E.g., if comorbid disease would be associated with overall survival through other-cause death rather than cancer-specific death, a wait and watch strategy or low-intensity treatment may be the best options. In this nationwide population-based register study, we examined the impact of severe comorbid disease history (according to the Charlson comorbidity index [19] and later modification [20]) on survival among patients diagnosed with myeloid leukemia or myeloma in Sweden 2002 to 2009. We also aimed to investigate potential variation in the effect of comorbidities on survival by type of comorbid disease, type of hematological malignancy and age.

## Methods

In a prospective register-based cohort study, we identified all individuals aged > 18 years, diagnosed with a first incident AML, chronic myeloid leukemia (CML) or myeloma from 2002 to 2009 in the Swedish Cancer Register (coverage > 95 % [21, 22]) using the International Classification of Diseases (ICD), 10^th^ revision (Additional file 1, Table S1). Patients diagnosed at autopsy or with a history of stem cell or solid organ transplantation prior to the leukemia/myeloma diagnosis (*n* = 47) were excluded. The study was approved by the Regional Ethical Committee in Stockholm, Sweden (2010/1624–32). Since we used de-identified register data, individual informed consent was not sought in line with institutional regulations.

### Comorbid disease

The cohort was linked to the Swedish Patient Register including in- and outpatient data (coverage 85–95 % [23]) to collect dates of hospital visits and admissions, and main and secondary diagnoses of comorbid disease listed in the modified Charlson index [19, 20] with the addition of psychiatric disorders (Additional file 1: Table S1), during a period of 5 years prior to the diagnosis of leukemia/myeloma. Records of rheumatologic and renal diseases were disregarded if they occurred during the year leading up to the diagnosis of leukemia/ myeloma since their occurrence could be a sign of the yet undiagnosed malignancy. Rheumatologic disorders only recorded closely in time to a diagnosis of a hematological malignancy may represent misclassified paramalignant phenomena rather than true autoimmune/inflammatory disease [24, 25]. Similarly, records of kidney dysfunction shortly before myeloma diagnosis could be a sign of myeloma rather than kidney disease [26]. Cancer history (excluding non-melanoma skin neoplasms) any time prior to the leukemia/myeloma was identified in the Swedish Cancer Register. Due to the possibility of misclassification between subtypes of hematological malignancies we excluded patients with prior history of any hematological malignancy from the study cohort. For patients with AML, we specifically noted a preceding diagnosis of myelodysplastic disorders (MDS) or myeloproliferative neoplasms (MPN).

### Sociodemographic factors

Through the longitudinal integrated database for health insurance and labor market (LISA), we assembled information on educational level [27]. The highest achieved educational level (< 10 years/10–12 years/ > 12 years) before diagnosis of leukemia/myeloma was used as a proxy for socioeconomic status [28, 29].

### Outcome

Patients were followed from the date of diagnosis of leukemia/myeloma until emigration, death or December 31st 2012, whichever occurred first. Dates and causes of death were obtained from the Cause-of-Death register (coverage > 99 % [30]). Death records with ICD10 codes for leukemia/myeloma as the main underlying cause of death were treated as cancer-specific death, otherwise as other-cause death (Additional file 1: Table S1). Leukemia- myeloma-specific death was defined along the lines proposed by Howlader et. al. [31] including a group of related codes for cancer-specific death. In validation studies, the information on main cause of death collected from Swedish Cause of Death Register for malignant neoplasms has been highly accurate [30, 32].

### Statistical methods

The associations of comorbid disease history and specific comorbidities with all-cause, cancer-specific and other-cause death were estimated as mortality rate ratios (MRR) with 95 % confidence intervals (CI) using Poisson regression. When estimating the effect of specific comorbid diseases on survival, all patients without the investigated type were included in the reference group. All analyses were adjusted for follow-up time (1-year intervals), age at diagnosis (10-year intervals), sex, calendar year of diagnosis, country of birth and education level. When assessing the statistical significance of interaction terms between comorbidity and time since diagnosis, we found no evidence of non-proportional hazards (*p* < 0.05). Moreover, for patients aged 60–89 years at diagnosis, the probability of cancer-specific and other-cause death was estimated in the presence of competing risks, using estimates from flexible parametric survival models [33]. These models use restricted cubic splines to model the baseline cause-specific hazard rates. The fitted models were stratified by age and sex, and used 3°-of-freedom to model the baseline hazard functions. All statistical analyses were performed with STATA software version 13 (StataCorp. 2013. College Station, TX: StataCorp LP).

## Results

We identified 2,550 patients with AML, 1,000 with CML and 4,584 with myeloma diagnosed in Sweden between 2002 and 2009 (Table 1). Median age at diagnosis was 72 years in AML and myeloma, and 67 years in CML. Median follow-up in AML was 0.6 (range 0–11) years, in CML 4.2 (range 0–11) years, and in myeloma 3.1 (range 0–11) years. Approximately 40 % of the patients had a history of comorbid disease (AML: 43 %; CML: 35 %; myeloma: 38 %) (Table 1). As expected, the prevalence of comorbidity increased with age and among patients diagnosed at 80+ years, more than half had a history of comorbidity (AML and CML: 59 %, myeloma 52 %). Non-hematological cancers (13–15 %) and cardiovascular disease (10–14 %) were the most common comorbid disease groups (Table 1).

**Table 1:** Characteristics of patients with AML, CML and myeloma, Sweden 2002–2009, and proportion with comorbid disease

	AML^a^		CML^b^		Myeloma	
	All	CD^c^	All	CD^c^	All	CD^c^
	No (%)	No (%)	No (%)	No (%)	No (%)	No (%)
Total	2550 (100)	1105 (43)	1000 (100)	350 (35)	4584 (100)	1735 (38)
Median follow up (year)	0.6 (0.0–11)		4.2 (0.0–11)		3.1 (0.0–11)	
*Sex*						
Women	1262 (50)	532 (42)	446 (45)	155 (35)	2075 (45)	745 (36)
Men	1288 (50)	573 (44)	554 (55)	195 (35)	2509 (55)	990 (39)
*Age*						
18–49	336 (13)	50 (15)	210 (21)	19 (9)	219 (4.8)	32 (15)
50–59	307 (12)	82 (27)	147 (15)	26 (18)	605 (13)	121 (20)
60–69	505 (20)	204 (40)	208 (21)	72 (35)	1145 (25)	349 (30)
70–79	746 (29)	382 (51)	242 (24)	120 (50)	1499 (33)	651 (43)
80+	656 (26)	387 (59)	193 (19)	113 (59)	1116 (24)	582 (52)
Median age (range)	72 (18–100)		67 (18–99)		72 (24–97)	
*Country of birth*						
Sweden	2279 (89)	996 (44)	881 (88)	313 (36)	4088 (89)	1553 (38)
Outside of Sweden	271 (11)	109 (40)	119 (12)	37 (31)	496 (11)	182 (37)
*Education level*						
0–9 years	1065 (42)	533 (50)	376 (38)	163 (43)	1997 (44)	888 (44)
10–12 years	958 (38)	383 (40)	400 (40)	122 (31)	1612 (35)	557 (35)
> 12 years	464 (18)	157 (34)	204 (20)	57 (28)	897 (20)	264 (29)
Missing	63 (2.5)	32 (51)	20 (2.0)	8 (40)	78 (1.7)	26 (33)
*No. of comorbid diseases*						
0	1445(57)		650 (65)		2849 (62)	
1	732 (29)		225 (22)		1154 (25)	
2+	373 (15)		125 (12)		581 (13)	
*Types of comorbid diseases*						
Cancer	372 (15)		134 (13)		601 (13)	
Cardiovascular disease	355 (14)		105 (10)		510 (11)	
Diabetes	235 (9.2)		72 (7.2)		377 (8.2)	
Cerebrovascular disease	200 (7.8)		54 (5.4)		262 (5.7)	
Chronic pulmonary disease	148 (5.8)		51 (5.1)		243 (5.3)	
Peripheral vascular disease	77 (3.0)		30 (3.0)		97 (2.1)	
Peptic ulcer disease	51 (2.0)		30 (3.0)		110 (2.4)	
Rheumatologic disease	82 (3.2)		20 (2.0)		71 (1.5)	
Renal disease	22 (0.9)		5 (0.5)		55 (1.2)	
Liver disease	20 (0.8)		8 (0.8)		44 (1.0)	
Dementia	25 (1.0)		10 (1.0)		49 (1.1)	
Psychiatric disorders	29 (1.1)		16 (1.6)		73 (1.6)	
Hemiplegia/Paraplegia	5 (0.2)		3 (0.3)		31 (0.7)	
AIDS/HIV	0 (0.0)		0 (0.0)		2 (0.04)	

^a^*AML* acute myeloid leukemia, ^b^*CML* chronic myeloid leukemia, ^c^*CD* comorbid disease

Most deaths were classified as cancer-specific, especially in AML (Additional file 2: Table S2). In general, patients with a history of any of the specified comorbid diseases had an increased rate of all-cause death compared with patients without such history (Table 2). The relative rate of other-cause death was higher than that of cancer-specific death, although cancer-specific death was also significantly increased among patients with comorbid disease history compared to those without in AML and myeloma, and borderline significantly increased among CML patients. In addition, female sex and higher attained education level tended to be associated with a more favorable prognosis (Table 2). Adjustment for age in 5-year instead of 10-year intervals did not change the results.

**Table 2:** MRR^a^ for all-cause, cancer-specific and other-cause death among AML CML and myeloma patients, Sweden 2002–2009

	AML^b^ MRR (95 % CI)	CML^c^ MRR (95 % CI)	Myeloma MRR (95 % CI)
All-cause death			
*Comorbid disease*			
No	1.00	1.00	1.00
Yes	**1.39 (1.26–1.52)**	**1.64 (1.34–2.01)**	**1.40 (1.30–1.50)**
*No. of comorbid diseases*			
0	1.00	1.00	1.00
1	**1.25 (1.12–1.38)**	**1.41 (1.12–1.77)**	**1.29 (1.19–1.40)**
2+	**1.78 (1.57–2.02)**	**2.22 (1.71–2.88)**	**1.67 (1.51–1.85)**
*Sex*			
Men	1.00	1.00	1.00
Women	0.91 (0.84–1.00)	0.83 (0.69–1.02)	**0.89 (0.83–0.95)**
*Education level*			
0–9	1.00	1.00	1.00
10–12	0.98 (0.89–1.09)	1.09 (0.89–1.35)	0.97 (0.89–1.04)
> 12	**0.80 (0.70–0.91)**	0.93 (0.70–1.25)	**0.83 (0.75–0.92)**
Cancer-specific death			
*Comorbid disease*			
No	1.00	1.00	1.00
Yes	**1.27 (1.15–1.40)**	1.28 (0.96–1.70)	**1.17 (1.08–1.28)**
*No. of comorbid diseases*			
0	1.00	1.00	1.00
1	**1.16 (1.04–1.29)**	1.12 (0.81–1.55)	**1.12 (1.02–1.23)**
2+	**1.60 (1.39–1.83)**	**1.66 (1.13–2.44)**	**1.31 (1.15–1.48)**
*Sex*			
Men	1.00	1.00	1.00
Women	0.92 (0.84–1.01)	0.91 (0.70–1.20)	0.91 (0.84–0.99)
*Education level*			
0–9	1.00	1.00	1.00
10–12	0.97 (0.87–1.08)	1.19 (0.89–1.58)	0.96 (0.88–1.06)
> 12	**0.80 (0.69–0.91)**	0.72 (0.46–1.12)	**0.87 (0.78–0.97)**
Other-cause death			
*Comorbid disease*			
No	1.00	1.00	1.00
Yes	**2.64 (2.00–3.48)**	**2.14 (1.60–2.85)**	**2.22 (1.94–2.55)**
*No. of comorbid diseases*			
0	1.00	1.00	1.00
1	**2.22 (1.64–3.01)**	**1.80 (1.30–2.49)**	**1.91 (1.64–2.23)**
2+	**3.86 (2.72–5.47)**	**2.98 (2.07–4.30)**	**3.02 (2.53–3.62)**
*Sex*			
Men	1.00	1.00	1.00
Women	0.90 (0.69–1.16)	0.76 (0.58–1.01)	**0.82 (0.72–0.94)**
*Education level*			
0–9	1.00	1.00	1.00
10–12	1.07 (0.79–1.43)	1.00 (0.73–1.36)	0.97 (0.83–1.13)
> 12	0.85 (0.58–1.25)	1.17 (0.80–1.73)	**0.72 (0.58–0.88)**

^a^*MRR* mortality rate ratios adjusted for age (in 10 year intervals), country of birth, time since diagnosis, calendar year of diagnosis and number of comorbid diseases, sex and education level except when main effects of these factors were estimated, statistically significant results (p<0.05) are in bold. ^b^*AML* acute myeloid leukemia, ^c^*CML* chronic myeloid leukemia

### Acute myeloid leukemia (AML)

**Fig. 1:**
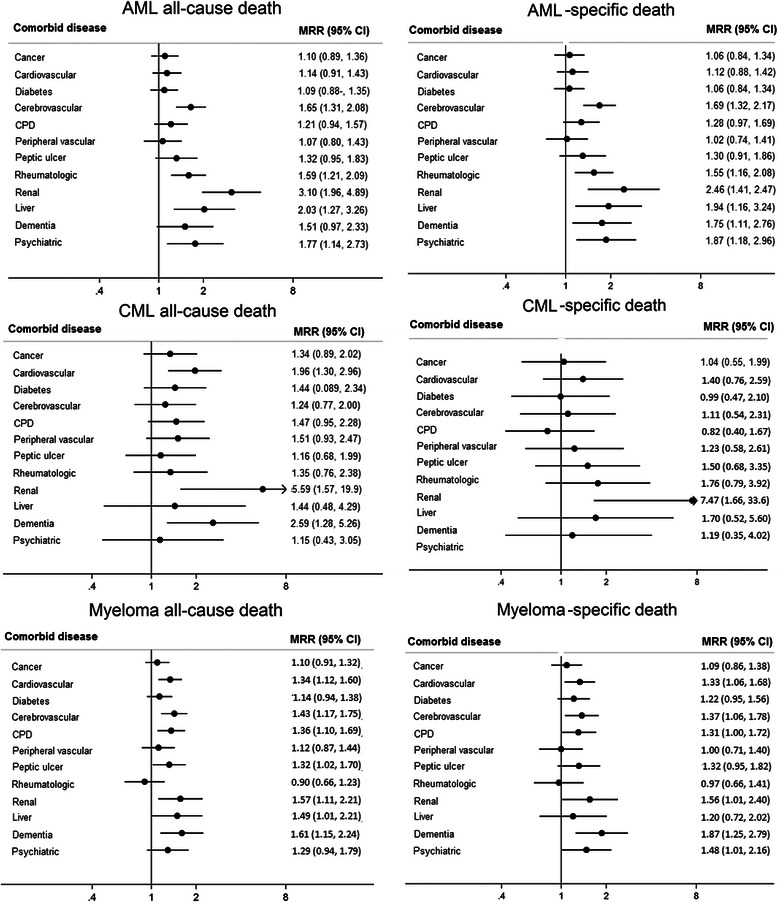
MRR for all-cause and cancer-specific death by type of comorbid disease. MRR mortality rate ratios adjusted for age (in 10 year intervals), country of birth, time since diagnosis, calendar year of diagnosis and number of comorbid diseases, sex and education level except when main effects of these factors were estimated. *AML* acute myeloid leukemia, *CML* chronic myeloid leukemia, *CPD***,** chronic pulmonary disease. ^*^Because of few patients with hemiplegia/paraplegia (*n* = 49) and HIV/AIDS (*n* = 2) overall, and with liver disease in CML, results for these groups are not presented

A higher all-cause as well as cancer-specific mortality in AML was observed for patients with previous cerebrovascular disease, rheumatologic diseases, renal disease, liver disease and psychiatric disease (Fig. 1). Dementia was also significantly associated with AML-specific mortality. Renal disorders were associated with the highest increase in mortality (MRR _all-cause death_ = 3.10, 95 % CI: 1.96–4.89; MRR _AML-specific death_ =2.46, 1.41–4.27, Fig. 1). Two-hundred and fourteen AML patients (8.3 %) had a prior record of MDS/MPN (MDS = 137, MPN = 77). Adjustment for previous MDS/MPN did not meaningfully alter the associations between non-hematological comorbidities and cancer-specific mortality. To address the relative contribution of prior cancer treatment, we also analyzed outcomes in association with non-malignant comorbidities separately, and results remained virtually unchanged.

**Fig. 2:**
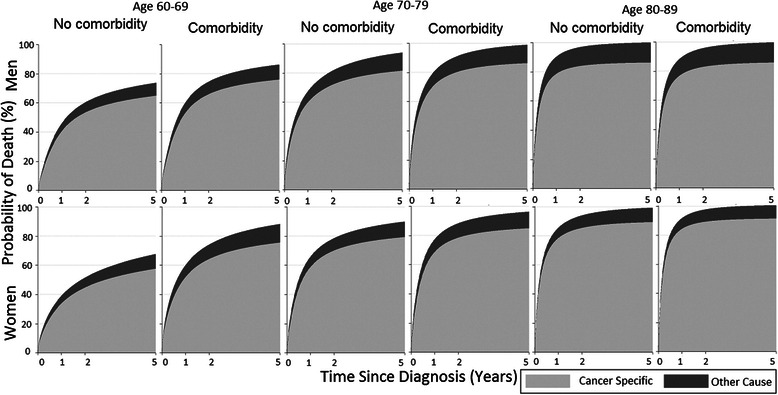
Stacked cumulative probability of cancer-specific and other-cause death among AML patients aged 60–89 years

In analyses of the absolute impact of comorbid disease history in the age groups 60–69, 70–79 and 80–89 years by sex, the probability of dying from AML was greater than the probability of dying from other causes in both sexes and in all investigated age groups, irrespective of the presence of comorbid disease (Fig. 2, Additional file 3: Table S3). The proportion of male patients aged 60–79 years who died from AML within the first 5 years after diagnosis was significantly higher for patients with at least one comorbid disease than for those without (ages 60–69: 76 % vs 65 %, difference 11 % (95 % CI 3.5–19); ages 70–79: 86 % vs 81 %, difference 4.8 % (95 % CI 1.5–7.9)) . Among patients 80–89 years, comorbid disease history was not associated with a higher cancer-specific probability of death (Fig. 2). For female patients aged 60–89 years, the pattern was similar, although in the oldest group, AML-specific deaths encompassed a larger share of all deaths as compared to males (Fig. 2, Additional file 4: Table S4).

### Chronic myeloid leukemia (CML)

**Fig. 3:**
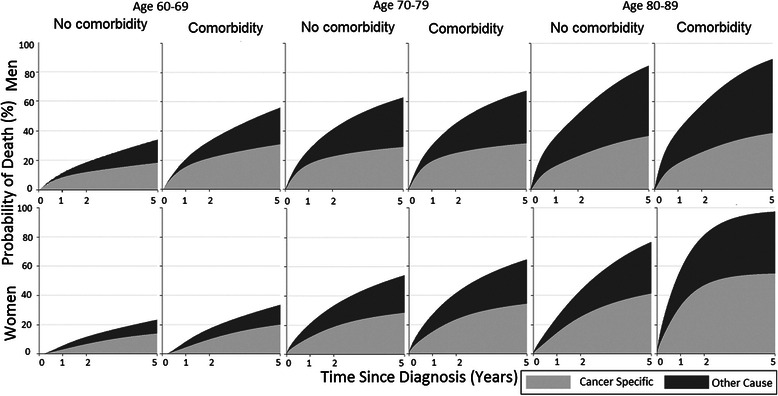
Stacked cumulative probability of cancer-specific and other-cause death, among CML patients aged 60–89 years

In analyses of specific comorbid diseases, most tended to be associated with a nominally higher all-cause as well as CML-specific mortality, but numbers were low reducing the precision. History of cardiovascular and renal disorders and dementia were significantly associated with all-cause death, whereas only renal disorders were associated with increased risk of CML-specific death (MRR = 7.47, 95 % CI: 1.66–33.6) (Fig. 1). Among men 70–89 years of age (but not those aged 60–69 years), the probability of dying from causes other than CML was greater than the probability of dying from CML within 5 years after diagnosis, regardless of the presence or absence of comorbidity (Fig. 3). Among men 60–69 years, the 5-year probability of CML-specific death was significantly higher for those with comorbid disease than those without (31 vs 18 %, difference 12.6 %, 95 % CI: 2.5–22.7, Additional file 3: Table S3). In older age groups there were no statistically significant differences in probabilities of cancer-specific or other-cause death among patients with and without comorbidities. In contrast, among women, comorbid disease conferred a higher probability of mainly cancer-specific death in ages 80–89 years (55 vs 41 %, difference 13.7, 95 % CI: 3.6–23.8) but no significant differences in cancer-specific or other-cause death in younger age groups (Additional file 4: Table S4).

### Myeloma

A history of cardiovascular, cerebrovascular, chronic pulmonary or renal disease, or dementia were associated with a higher all-cause as well as myeloma-specific mortality (Fig. 1). Liver and ulcer disease were additionally associated with all-cause mortality whereas psychiatric disease was associated with myeloma-specific mortality only. No one with renal disease had a complementary diagnosis code of amyloidosis.

**Fig. 4:**
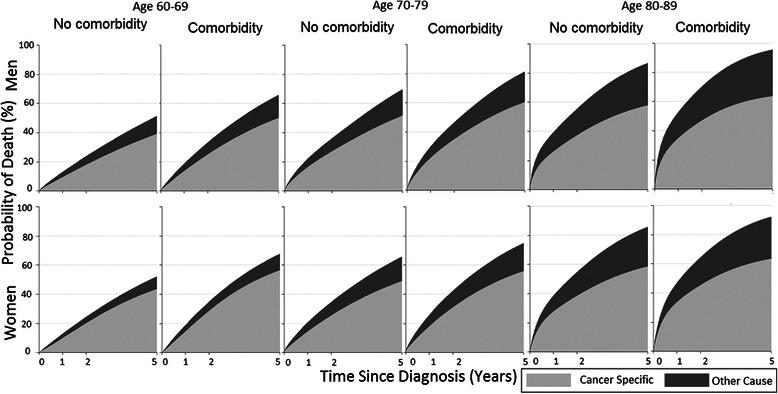
Stacked cumulative probability of cancer-specific and other-cause death, among myeloma patients aged 60–89 years

All myeloma patient groups by sex and age (60–89 years) were more likely to die of myeloma than other causes (Fig. 4). Irrespective of age at diagnosis and sex, the 5-year probability of death from myeloma mainly, but also of other-cause death, was significantly higher among patients with comorbidities than among patients without (Additional file 3: Table S3, Additional file 4: Table S4). In absolute terms, the percentage differences in the probabilities of patients who died from myeloma within 5 years in the two groups with and without comorbidity were moderate, ranging from 5.8 to 10.9 (Fig. 4).

## Discussion

In this large population-based study, using prospectively recorded information on comorbid diseases, we showed that patients with a history of comorbidity at diagnosis of AML, CML or myeloma had a higher all-cause but also cancer-specific mortality compared with patients without such history, reflecting an impact on disease-specific outcome in these malignancies. Renal disorders were associated with a markedly higher cancer-specific mortality among all three patient groups (but were uncommon, prevalence 0.5–1 %). Cerebrovascular disease, dementia and psychiatric disease were associated with an increased risk of cancer-specific death in AML and myeloma patients, liver and rheumatologic disease increased risk in AML only, and cardiovascular and chronic pulmonary disease in myeloma only. In absolute terms, the 5-year probability of cancer-specific death was greater than that of other-cause death among all patients aged 60–89 years except among male patients > 70 years with CML. Comorbidity contributed most to cancer-specific death among younger patients (< 70 years) in AML, whereas the impact was constant by age in myeloma.

### AML

The achievement of complete remission and long-term survival in AML mostly requires intensive combination chemotherapy, and outcomes are strongly dependent upon age and performance status [34, 35]. During the study period in Sweden, the majority of the patients diagnosed up to the age of 80 years received intense treatment [34]. Whether further prognostic stratification and personalized therapy can be achieved by adding a more systematic evaluation of comorbidity has been investigated in a few previous studies. In most [12, 13, 36–38], but not all [14] of these, comorbidity assessed using the Charlson index was independently associated with a worse overall survival. Etienne et al. (*N* = 133) showed that comorbid diseases (with an index score > 1) negatively predicted complete remission rate [13]. In two recent large studies, a lower likelihood of treatment with intense chemotherapy was noted in the presence of comorbid disease [14, 37]. In Ostgard et al. [14], comorbidity was not associated with survival when adjusted for performance status and other factors. Performance status could however be considered an intermediate explanatory factor rather than a true confounder and therefore an association between comorbidity and survival through lower performance status is still plausible. In that study, outcome was also investigated in relation to specific comorbid disease, and dementia, heart failure and renal failure were associated with opting-out of intensive therapy [14]. This is in line with our findings of a decreased cancer-specific survival in patients with renal disease and dementia. A similar explanation is plausible among patients with cerebrovascular and psychiatric disease, also noted to have a worse cancer-specific survival in our study.

Ostgard et al. also observed an indication of a stronger association between comorbidity and outcome among patients < 60 versus > 60 years of age. Extending these previous results, we show a clear differential effect of comorbidity by age with a larger prognostic importance of comorbidity among patients 60–69 years versus older patients. Hence, our results provide additional support for the notion that poor outcomes among AML patients > 70 years cannot be explained solely on the basis of increased prevalence of comorbidity by age but [14, 34], rather through a more general low treatment tolerance at older ages.

### CML

CML survival has improved greatly with the introduction of tyrosine kinase inhibitors such as imatinib (introduced in 2001 in Sweden) [39] although elderly Swedish CML patients (> 79 years) still have a 5-year relative survival of only 60 % [40], that may reflect under-implementation of tyrosine kinase inhibitor use [39, 40]. A few previous studies have shown a negative impact of comorbidity on CML survival in line with our results, mainly reflected in a poorer event-free survival [36] or lower degree of complete cytogenic response [41, 42]. Previous studies have assessed comorbidity through pooled indices including the Charlson comorbidity index [36, 41], or the adult comorbidity evaluation-27 score and cumulative illness rating scale [36, 41], but have not investigated survival by specific comorbidities, perhaps due to low numbers. We show for the first time that prior renal disease is associated with a poorer cancer-specific survival in CML. Renal disease is not an absolute contraindication for use of tyrosine kinase inhibitors, but glomerular filtration rate may decrease further during tyrosine kinase inhibitors treatment [43]. Thus, dose reductions [44] or caution to prescribe tyrosine kinase inhibitors could potentially explain this finding. Also, high comorbidity index has been associated with an increased risk of toxicity to tyrosine kinase inhibitors in two previous studies [41, 45].

A previous cohort study indicated that treatment of elderly CML patients (*n* = 181, median age 79 years) might be influenced by the individual physician’s perception and could be improved by utilizing comorbidity indices [36]. In our study, comorbidities were only associated with a higher probability of CML-specific death among men 60–69 years of age but not among older patients. Among the elderly males (> 70 years of age), other-cause deaths outweighed CML-specific deaths regardless of comorbidity. In contrast, among women with CML, comorbidity was only associated with a higher probability of CML-specific death in the oldest group (80+). Women with CML were more likely to die of CML-specific rather than other-cause death up to 89 years. Previous Swedish studies have noted a possible reluctance to treat elderly patients with tyrosine kinase inhibitors during the investigated time-period [39, 46]. The present findings indicate that CML outcome could potentially be further improved among elderly, especially female patients.

### Myeloma

Survival in multiple myeloma has increased during recent decades especially among younger patients (< 60-70 years), likely due to a combination of factors including increasing use of high-dose Melphalan with stem cell support and thalidomide as well as improvements in supportive care [47, 48]. Previous studies have reported comorbidities to be of critical prognostic importance at myeloma diagnosis using different comorbidity indices [49, 50]. In particular, renal impairment (pre-existing or disease-related) has been identified as an important determinant for myeloma outcome [50, 51]. Kleber et al. have developed the Freiburg comorbidity index including performance status, renal impairment and lung disease, and have reported large differences in overall survival among 466 myeloma patients (median age 62 years) by the presence or absence of a combination of these factors [49]. In our study including ~ 4,500 myeloma patients with a median age of 72 years, we confirm the adverse prognostic impact of pre-existing renal and pulmonary disease, and extend the list of disorders associated with a higher risk of cancer-specific death to also include cardiovascular and cerebrovascular disease, dementia and psychiatric disorders. Hence, our study suggests that future evaluations of comorbidity and myeloma outcome in larger studies may benefit from including a broader list of disorders [52]. Interestingly, and in contrast with AML and CML, the prognostic impact of comorbid disease seemed relatively constant by age (among patients aged 60–89 years) [53].

The strengths of our study include the large size of the cohort, the high quality and coverage of the registers used as well as the population-based unselected setting, evaluating the effect of 12 severe comorbid diseases on outcome of hematological malignancies in the most recent decade. We also, for the first time in this setting, used a novel methodology to estimate probabilities of death associated with comorbidities in patients with hematological malignancies, in the presence of competing risks. While traditional ratio estimates of net survival (such as those presented in Table 2) are important to identify and evaluate the impact of prognostic factors associated with the disease under study, competing risks analyses may provide additional insights to understanding the real-world prognosis of the patients. This is because, in contrast to estimates of net survival, a competing risks analysis takes into account that causes other than the malignancy may kill the patient first and thereby preclude death from the malignancy. Thus, competing risks analyses more appropriately reflect the absolute impact of a prognostic factor on prognosis [54]. The advantage of studying the three different hematological malignancies together was to contrast between malignancy types needing intensive treatment upfront (AML) and those with slower tumor progression in need of more intermediate-intensity treatment (myeloma, CML). An important limitation of our study was the lack of clinical data such as performance status, disease-specific prognostic determinants including genetic abnormalities, and treatment. Since prior comorbidity may lead to lower performance status and may affect choices of treatment, rather than the other way around, performance status and treatment intensity may be considered explanatory factors rather than true confounders when estimating the impact of comorbidity on survival. Another limitation to consider is the definition of deaths as cancer-specific. Although the accuracy of the classification of main underlying cause of death has been found to be high for malignant diseases in the Swedish Cause-of-death registers [30, 32], some leukemia/myeloma deaths may have been erroneously classified as non-cancer-related or vice versa. However, given that the majority of the deaths were cancer-specific and that we also present patterns of all-cause and other-cause deaths, a minor degree of such misclassification does not threaten our main conclusions.

## Conclusion

Patients with AML, CML and myeloma have a high prevalence of comorbid disease especially in older ages. In the present study, comorbidities associated with worse cancer-specific mortality primarily included diseases associated with organ failure and with reduced cognitive function. Several comorbid diseases were associated with higher AML-specific and myeloma-specific mortality, whereas in CML, only renal disease was associated with a worse cancer-specific outcome. The impact of comorbidity varied by age and was most pronounced among AML patients younger than 70 years. Cancer-specific deaths outnumbered other-cause deaths in all patient groups except male patients with CML above 70 years of age. The results highlight the need for clinical awareness around comorbid patient groups and patient information, as well as an urgent need for the development and evaluation of alternative effective but less toxic treatment regimens.

## Additional files

Additional file 1: **Table S1.** International Classification of Disease (ICD) codes, 10th revision, for classification of leukemia/myeloma, and comorbid diseases. (DOCX 13 kb)
Additional file 2: **Table S2.** Number of outcome events and event rate overall and by type of comorbid disease. (DOCX 13 kb)
Additional file 3: **Table S3.** Probabilities of death at 1, 2 and 5 years of follow-up among men aged 60–89 years. (DOCX 14 kb)
Additional file 4: **Table S4.** Probabilities of death at 1, 2 and 5 years of follow-up among women aged 60–89 years. (DOCX 14 kb)
