# Supplementary material for: The impact of comorbid disease history on all-cause and cancer-specific mortality in myeloid leukemia and myeloma – a Swedish population-based study
Source: BMC Cancer. 2015 Nov 5;15:850. doi: 10.1186/s12885-015-1857-x (PMC4634819; doi:10.1186/s12885-015-1857-x)
Supplement: Additional file 1: — Table S1. International Classification of Disease (ICD) codes, 10th revision, for classification of leukemia/myeloma, and comorbid diseases. (DOCX 13 kb) [file 12885_2015_1857_MOESM1_ESM.docx]

**Table S1.** International Classification of Disease (ICD) codes, 10^th^ revision, for classification of leukemia/myeloma, and comorbid diseases.

|  | **Diagnosis of malignancy** | **Cancer-specific death** |
| --- | --- | --- |
| **Malignancy types** | |  |
| Acute myeloid leukemia | C920, C922- C925, C930, C940, C942 | C920, C922- C925, C930, C937, C939, C940, C927, C929, C933, C939, C943, C947, C950, C957, C959, |
| Chronic myeloid leukemia | C921, C927, C931, C932, C937 | C921, C927, C931, C932, C937, C927, C929, C933, C939, C943, C947, C950, C951, C957, C959 |
| Myeloma/plasmocytoma | C900-C902 | C900-C902 |
| **Comorbid disease** | | |
| Cancer | C00-C43, C45-C80, C97 | |
| Cardiovascular disorders |  | |
| Myocardial infarction | I21-I22, I252 | |
| Congestive Heart failure | I099, I110, I130, I132, I255, I420, I425-I429, I43, I50, P290 | |
| Diabetes | E10-E14 | |
| Cerebrovascular disease | G45-G46, H340, I60-I69 | |
| Chronic pulmonary disease | I278, I279, J40, J42-J47, J60-J67, J684, J701, J703 | |
| Peripheral vascular disease | I70-I71, I731, I738-I739, I771, I790, I792, K551, K558-K559, Z958-Z959 | |
| Peptic ulcer disease | K25-K28 | |
| Rheumatologic disease | M05-M06, M315, M32-M34, M351,M353, M360 | |
| Renal disease | I120, I131, N032-N037, N052-N057, N18-N19, N250, Z490, Z492, Z992 | |
| Liver disease | B18, I850, I859, I864, I982, K700-K704, K709, K711, K713, K715, K717, K721, K729, K73-K74, K760, K762, K764-K769 | |
| Dementia | F00-F03, F051, G30, G311 | |
| Psychiatric disorders | F10-F14, F16, F18-F19, F20, F22-25, F28-F29, F302, F312, F315 | |
| Hemiplegia/paraplegia | G041, G114, G801, G802, G81, G82, G830-G834, G839 | |
| HIV/AIDS | B20-B22, B24 | |
