# Supplementary material for: The impact of comorbid disease history on all-cause and cancer-specific mortality in myeloid leukemia and myeloma – a Swedish population-based study
Source: BMC Cancer. 2015 Nov 5;15:850. doi: 10.1186/s12885-015-1857-x (PMC4634819; doi:10.1186/s12885-015-1857-x)
Supplement: Additional file 2: — Table S2. Number of outcome events and event rate overall and by type of comorbid disease. (DOCX 13 kb) [file 12885_2015_1857_MOESM2_ESM.docx]

**Table S2.** Number of outcome events and event rate overall and by type of comorbid disease

|  | AML | | | | CML | | | | Myeloma | | | |
| --- | --- | --- | --- | --- | --- | --- | --- | --- | --- | --- | --- | --- |
|  | All-cause death | | AML-specific death | | All-cause death | | CML-specific death | | All-cause death | | Myeloma-specific death | |
| Comorbid disease history | No | Rate^1^ | No | Rate^1^ | No | Rate^1^ | No | Rate^1^ | No | Rate^1^ | No | Rate^1^ |
| No comorbid disease | 1080 | 3.0 | 992 | 2.8 | 224 | 0.7 | 128 | 0.4 | 1926 | 1.8 | 1530 | 1.4 |
| Cancer | 351 | 9.3 | 292 | 7.7 | 96 | 2.4 | 43 | 1.1 | 499 | 3.1 | 323 | 2.0 |
| Cardiovascular | 334 | 10.9 | 278 | 9.0 | 91 | 4.5 | 39 | 1.9 | 443 | 4.0 | 281 | 2.5 |
| Diabetes | 217 | 7.9 | 175 | 6.4 | 46 | 2.0 | 18 | 0.8 | 303 | 2.9 | 208 | 2.0 |
| Cerebrovascular | 189 | 11.9 | 164 | 10.3 | 34 | 2.1 | 16 | 1.0 | 232 | 4.2 | 143 | 2.6 |
| CPD | 137 | 10.0 | 121 | 8.9 | 39 | 2.8 | 12 | 0.9 | 206 | 3.4 | 127 | 2.1 |
| Peripheral Vascular | 74 | 13.8 | 59 | 11.0 | 23 | 2.6 | 10 | 1.1 | 81 | 3.5 | 46 | 2.0 |
| Peptic ulcer | 47 | 9.4 | 39 | 7.8 | 19 | 2.2 | 10 | 1.2 | 97 | 3.9 | 62 | 2.5 |
| Rheumatologic | 79 | 15.2 | 67 | 12.9 | 15 | 2.6 | 8 | 1.4 | 52 | 2.5 | 35 | 1.7 |
| Renal | 22 | 57.4 | 16 | 41.7 | 4 | 5.9 | 3 | 4.4 | 45 | 3.7 | 29 | 2.4 |
| Liver | 20 | 17.6 | 17 | 14.9 | 4 | 1.7 | 0 | 0 | 32 | 2.4 | 18 | 1.4 |
| Dementia | 25 | 23.0 | 24 | 22.1 | 10 | 8.3 | 3 | 2.5 | 45 | 5.6 | 33 | 4.1 |
| Psychiatric | 25 | 8.6 | 23 | 7.9 | 7 | 1.2 | 5 | 0.8 | 56 | 2.5 | 42 | 1.9 |

^1^Per 10 person-years
