# Supplementary material for: The impact of comorbid disease history on all-cause and cancer-specific mortality in myeloid leukemia and myeloma – a Swedish population-based study
Source: BMC Cancer. 2015 Nov 5;15:850. doi: 10.1186/s12885-015-1857-x (PMC4634819; doi:10.1186/s12885-015-1857-x)
Supplement: Additional file 3: — Table S3. Probabilities of death at 1, 2 and 5 years of follow-up among men aged 60–89 years. (DOCX 14 kb) [file 12885_2015_1857_MOESM3_ESM.docx]

**Table S3.** Probabilities of death at 1, 2 and 5 years of follow-up **among men** aged 60-89 years

| Age | 60-69 | | 70-79 | | 80-89 | |
| --- | --- | --- | --- | --- | --- | --- |
|  | Comorbid disease | | Comorbid disease | | Comorbid disease | |
| Follow up (years) | No/Yes | Difference ^1^ (%, 95%CI) | No/Yes | Difference ^1^ (%, 95%CI) | No/Yes | Difference ^1^ (%, 95%CI) |
| **AML** |  |  |  |  |  |  |
| AML-specific death | | |  |  |  |  |
| 1 | 40.7/52.7 | **12.0 (3.5-20.5)** | 59.9/70.4 | **10.5 (4.2-16.7)** | 78.5/76.5 | -1.7 (-7.0-3.5) |
| 2 | 53.4/65.8 | **12.4 (3.8-21.0)** | 71.6/80.0 | **8.4 (3.3-13.6)** | 83.8/82.8 | -1.0 (-4.1-2.1) |
| 5 | 64.8/75.8 | **11.0 (3.5-18.6)** | 81.4/86.2 | **4.8 (1.5-7.9)** | 86.2/85.7 | -0.4 (-1.8-0.9) |
| Other cause death | | |  |  |  |  |
| 1 | 5.3/6.9 | **1.6 (0.3-3.0)** | 7.5/9.1 | **1.6 (0.5-2.8)** | 11.7/11.4 | -0.3 (-1.1-0.6) |
| 2 | 6.9/8.5 | **1.6 (0.3-3.0)** | 9.4/10.7 | **1.3 (0.3-2.2)** | 12.9/12.8 | -0.1 (-0.5-0.3) |
| 5 | 8.8/10.2 | **1.4 (0.2-2.6)** | 12.3/12.5 | 0.2 (-0.7-1.0) | 13.6/13.7 | 0.1 (-0.3-0.5) |
| **CML** | |  |  |  |  |  |
| CML-specific death | | |  |  |  |  |
| 1 | 8.2/15.4 | **7.2 (0.8-13.6)** | 17.2/19.2 | 2.0 (-4.3-8.2) | 15.5/17.8 | 2.3 (-3.3-7.8) |
| 2 | 11.7/21.4 | **9.6 (1.5-17.7)** | 22.4/24.7 | 2.3 (-5.2-9.8) | 22.6/25.3 | 2.7 (-4.0-9.5) |
| 5 | 18.1/30.7 | **12.6 (2.5-22.7)** | 28.9/31.4 | 2.5 (-5.5-10.6) | 36.4/38.4 | 2.0 (-3.1-7.1) |
| Other cause death | | |  |  |  |  |
| 1 | 2.9/5.3 | 2.4 (-0.3-5.2) | 9.8/10.8 | 1.1 (-2.4-4.5) | 19.5/22.4 | 2.9 (-4.3-10.2) |
| 2 | 6.6/11.5 | **4.9 (0.4-9.5)** | 18.4/20.1 | 1.7 (-3.7-7.1) | 28.7/32.2 | 3.5 (-5.2-12.3) |
| 5 | 15.8/25.0 | **9.2 (1.8-16.6)** | 33.8/35.9 | 2.1 (-4.6-8.8) | 48.0/50.5 | 2.5 (-3.9-8.9) |
| **Myeloma** | |  |  |  |  |  |
| Myeloma-specific death | | |  |  |  |  |
| 1 | 9.4/13.5 | **4.1 (1.9-6.4)** | 16.6/22.2 | **5.6 (3.0-8.2)** | 26.1/34.8 | **8.7 (4.7-12.7)** |
| 2 | 17.9/25.0 | **7.1 (3.4-10.9)** | 27.0/34.9 | **7.9 (4.3-11.5)** | 37.1/46.9 | **9.7 (5.3-14.2)** |
| 5 | 39.0/49.9 | **10.9 (5.5-16.5)** | 51.5/60.3 | **8.8 (4.8-12.8)** | 57.7/63.5 | **5.8 (2.9-8.8)** |
| Other cause death | | |  |  |  |  |
| 1 | 3.5/5.1 | **1.6 (0.6-2.5)** | 5.8/7.7 | **2.0 (1.0-3.0)** | 13.2/17.8 | **4.6 (2.4-6.9)** |
| 2 | 5.8/8.2 | **2.4 (1.0-3.8)** | 8.9/11.5 | **2.6 (1.4-3.9)** | 18.1/23.2 | **5.1 (2.6-7.5)** |
| 5 | 12.2/15.7 | **3.5 (1.6-5.4)** | 18.0/20.9 | **2.9 (1.5-4.3)** | 28.9/31.9 | 3.0 (-1.5-1.9) |

^1^ Difference (95%CI) = Difference between probability of death in patients with comorbid disease and without comorbid disease with 95 confidence interval. Statistically significant results (p<0.05) are in bold.
