# Supplementary material for: The impact of comorbid disease history on all-cause and cancer-specific mortality in myeloid leukemia and myeloma – a Swedish population-based study
Source: BMC Cancer. 2015 Nov 5;15:850. doi: 10.1186/s12885-015-1857-x (PMC4634819; doi:10.1186/s12885-015-1857-x)
Supplement: Additional file 4: — Table S4. Probabilities of death at 1, 2 and 5 years of follow-up among women aged 60–89 years. (DOCX 14 kb) [file 12885_2015_1857_MOESM4_ESM.docx]

**Table S4.** Probabilities of death at 1, 2 and 5 years of follow-up **among women** aged 60-89 years

| Age | 60-69 | | 70-79 | | 80-89 | |
| --- | --- | --- | --- | --- | --- | --- |
| Follow up (years) | Comorbid disease | | Comorbid disease | | Comorbid disease | |
|  | No/Yes | Difference ^1^ (95%CI) | No/Yes | Difference ^1^ (95%CI) | No/Yes | Difference ^1^ (95%CI) |
| AML | |  |  |  |  |  |
| AML-specific death | | |  |  |  |  |
| 1 | 33.8/52.1 | **18.3 (9.4-27.2)** | 58.9/68.8 | **10.0 (3.1-16.9)** | 78.5/84.3 | **5.9 (0.0-11.8)** |
| 2 | 44.7/64.3 | **19.6 (10.2-29)** | 70.0/78.4 | **8.4 (2.6-14.2)** | 85.4/89.2 | 3.8 (-0.1-7.8) |
| 5 | 57.5/75.4 | **17.9 (9.3-26.4)** | 79.2/84.8 | **5.6 (1.6-9.6)** | 89.3/91.3 | 2.0 (-0.3-4.3) |
| Other cause death | | |  |  |  |  |
| 1 | 5.2/8.2 | **3.0 (0.9-5.0)** | 7.0/8.6 | **1.6 (0.3-2.9)** | 7.0/7.3 | 0.3 (-0.2-0.8) |
| 2 | 6.7/9.9 | **3.2 (1.0-5.2)** | 8.4/9.8 | **1.4 (0.2-2.5)** | 8.5/8.4 | -0.2 (-0.7-0.4) |
| 5 | 10.0/12.6 | **2.6 (0.7-4.6)** | 10.5/11.2 | 0.7 (-0.2-1.7) | 9.8/9.1 | -0.7 (-1.9-0.4) |
| CML | |  |  |  |  |  |
| CML-specific death | | |  |  |  |  |
| 1 | 2.9/4.4 | 1.5 (-1.5-4.5) | 11.8/15.2 | 3.4 (-2.4-9.1) | 14.3/32.8 | **18.5 (9.3-27.7)** |
| 2 | 6.6/9.9 | 3.3 (-2.8-9.3) | 18.8/23.6 | 4.8 (-3.3-12.8) | 25.2/46.9 | **21.7 (11.0-32.4)** |
| 5 | 13.8/19.9 | 6.1 (-4.8-17.0) | 28.4/34.2 | 5.8 (-4.0-15.5) | 41.1/54.8 | **13.7 (3.6-23.8)** |
| Other cause death | | |  |  |  |  |
| 1 | 2.5/3.8 | 1.3 (-1.3-3.9) | 9.2/11.8 | 2.6 (-1.9-7.1) | 10.5/24.7 | **14.2 (6.3-22.0)** |
| 2 | 4.8/7.2 | 2.4 (-2.2-6.9) | 14.4/18.0 | 3.6 (-2.6-9.8) | 18.0/34.3 | **16.3 (7.5-25.0)** |
| 5 | 9.3/13.5 | 4.2 (-3.4-11.7) | 25.6/30.3 | 4.7 (-3.3-12.7) | 35.3/42.3 | 7.0 (-1.7-15.7) |
| Myeloma | |  |  |  |  |  |
| Myeloma-specific death | | |  |  |  |  |
| 1 | 9.8/14.5 | **4.7 (1.8-7.6)** | 14.4/18.0 | **4.7 (1.8-7.6)** | 27.6/33.7 | **6.1 (2.0-10.1)** |
| 2 | 20.0/28.5 | **8.5 (3.5-13.4)** | 25.4/31.0 | **5.6 (1.8-9.4)** | 38.4/45.3 | **6.9 (2.3-11.5)** |
| 5 | 43.4/56.2 | **12.8 (5.8-19.9)** | 48.8/55.7 | **6.9 (2.3-11.5)** | 58.4/63.3 | **4.9 (1.6-8.2)** |
| Other cause death | | |  |  |  |  |
| 1 | 2.9/4.4 | **1.4 (0.4-2.5)** | 6.2/7.8 | **1.4 (0.4-2.5)** | 10.9/13.3 | **2.4 (0.7-4.1)** |
| 2 | 4.3/6.2 | **1.9 (0.6-3.3)** | 9.2/11.3 | **2.1 (0.6-3.7)** | 16.2/19.0 | **2.8 (0.9-4.7)** |
| 5 | 8.6/11.2 | **2.6 (1.0-4.3)** | 16.7/19.2 | **2.5 (0.8-4.2)** | 27.1/28.7 | **1.6 (0.4-2.9)** |

^1^ Difference (95% CI) = Difference between probability of death in patients with comorbid disease and without comorbid disease with 95 confidence interval. Statistically significant results (p<0.05) are in bold.
